# Association of homocysteine with white matter dysconnectivity in schizophrenia

**DOI:** 10.1038/s41537-024-00458-0

**Published:** 2024-03-20

**Authors:** Koichi Tabata, Shuraku Son, Jun Miyata, Kazuya Toriumi, Mitsuhiro Miyashita, Kazuhiro Suzuki, Masanari Itokawa, Hidehiko Takahashi, Toshiya Murai, Makoto Arai

**Affiliations:** 1 Schizophrenia Research Project, Department of Psychiatry and Behavioral Sciences, Tokyo Metropolitan Institute of Medical Science, Tokyo, Japan; 2 Department of Psychiatry and Behavioral Sciences, Graduate School of Medical and Dental Sciences, Tokyo Medical and Dental University, Tokyo, Japan; 3 Department of Psychiatry, Graduate School of Medicine, Kyoto University, Kyoto, Japan; 4 Unit for Mental Health Promotion, Research Center for Social Science & Medicine, Tokyo Metropolitan Institute of Medical Science, Tokyo, Japan; 5 Department of Psychiatry, Shinshu University School of Medicine, Matsumoto, Japan; 6 Department of Psychiatry, Tokyo Metropolitan Matsuzawa Hospital, Tokyo, Japan; 7 Center for Brain Integration Research, Tokyo Medical and Dental University, Tokyo, Japan

**Keywords:** Schizophrenia, Biomarkers, Schizophrenia

## Abstract

Several studies have shown white matter (WM) dysconnectivity in people with schizophrenia (SZ). However, the underlying mechanism remains unclear. We investigated the relationship between plasma homocysteine (Hcy) levels and WM microstructure in people with SZ using diffusion tensor imaging (DTI). Fifty-three people with SZ and 83 healthy controls (HC) were included in this retrospective observational study. Tract-Based Spatial Statistics (TBSS) were used to evaluate group differences in WM microstructure. A significant negative correlation between plasma Hcy levels and WM microstructural disruption was noted in the SZ group (Spearman’s ρ = −.330, *P* = 0.016) but not in the HC group (Spearman’s ρ = .041, *P* = 0.712). These results suggest that increased Hcy may be associated with WM dysconnectivity in SZ, and the interaction between Hcy and WM dysconnectivity could be a potential mechanism of the pathophysiology of SZ. Further, longitudinal studies are required to investigate whether high Hcy levels subsequently cause WM microstructural disruption in people with SZ.

## Introduction

Recently, several reports have demonstrated white matter (WM) dysconnectivity in individuals with schizophrenia (SZ) across their lifespan, including during the first episode and acute and chronic phases^1–3^. Diffusion tensor imaging (DTI) is used to identify WM microstructural disruptions, revealing significantly lower fractional anisotropy (FA) in individuals with SZ than in healthy controls (HC). FA reductions in SZ have been identified in various brain regions, including the frontal lobe, thalamus, cingulate gyrus, and temporal lobe^1–3^. Furthermore, a multisite harmonization study demonstrated an association between FA reduction and core cognitive impairment, including language, processing speed, working memory, and nonverbal memory, which is known to impact real-world functioning in people with SZ^4–6^. These findings suggest global dysfunction in brain networks rather than localized disruption of specific brain regions and tracts. However, the underlying mechanism of WM dysconnectivity in SZ remains unclear.

Accumulating evidence has shown that homocysteine (Hcy) is involved in the pathophysiology of SZ^7–9^. A meta-analysis of genome-wide association studies showed a significant association between SZ and the methylenetetrahydrofolate reductase (*MTHFR*) C677T (rs1801133) polymorphism, which leads to reduced enzyme activity and increased plasma Hcy levels^7^. Furthermore, high Hcy levels in plasma have been suggested as a risk factor for SZ. A Mendelian randomization analysis yielded an odds ratio of 2.15 per 1-SD increase in plasma Hcy levels^10^. Additionally, several studies, including one conducted in drug-naïve, first-episode psychosis patients^11^, have reported that plasma Hcy levels are positively correlated with the clinical severity of SZ using the Positive and Negative Syndrome Scale (PANSS)^11–13^. Therefore, these findings implicate increased Hcy as a part of the biological etiology of SZ.

According to several in vitro and in vivo studies, exposure to Hcy disrupts the WM through some mechanisms: Hcy interacts with neuronal N-methyl-D-aspartate (NMDA) receptors, initiates oxidative stress, induces apoptosis, and leads to vascular damage^14,15^. Indeed, some reports have indicated an association between Hcy and WM dysconnectivity in older people with neuropsychiatric disorders, including depression^16^, Alzheimer’s disease^17^, and Parkinson’s disease^18^. However, the relationship between plasma Hcy levels and WM dysconnectivity in people with SZ remains unknown.

Thus, to better understand the pathophysiology of WM dysconnectivity in SZ, we examined the relationship between plasma Hcy levels and WM microstructural disruption using DTI in people with SZ.

## Methods

### Study design and participants

In this retrospective observational study, we reviewed the clinical data of the recruited participants as described previously^19^. A total of 53 people with SZ and 83 age- and sex-matched HC were recruited. Individuals met the criteria for SZ based on the Structural Clinical Interview for DSM-IV Axis I Disorders (SCID) patient edition, version 2.0 and had no history of other psychiatric disorders. Psychiatric symptoms were assessed using the Positive and Negative Syndrome Scale (PANSS). Individuals in the HC group were assessed using the SCID non-patient edition, version 2.0 and had no previous psychiatric disorders nor any first-degree relatives with a history of psychotic episodes. Exclusion criteria for both groups included a history of head trauma, neurological disease, severe medical conditions, or substance abuse that could affect brain function. Almost all people with SZ were receiving antipsychotic medication at the time of the study (none [*N* = 1], typical [*N* = 2], atypical [*N* = 41], typical and atypical [*N* = 5], unknown [*N* = 4]) (Supplementary Table 1). To assess the antipsychotic doses people with SZ actually received, chlorpromazine (CP) equivalents were calculated according to the Practice Guideline for the Treatment of Schizophrenia Patients^20,21^. The CP equivalent data were unavailable for 4 of the 53 people with SZ. Thus, we analyzed 49 people with SZ for all analyses using CP equivalents. Written informed consent was obtained from all participants. This study was approved by the Committee on Medical Ethics of Kyoto University and was conducted in accordance with the Code of Ethics of the World Medical Association.

### Measurement and group comparisons of plasma Hcy levels

Plasma samples were obtained from all participants on the same day as the MRI scans. All plasma Hcy measurements were conducted at SRL Inc. (Tokyo, Japan). Non-parametric analyses using the Mann–Whitney *U* test were performed to examine between-group differences in plasma Hcy levels. To explore the potential effects of age and sex on between-group differences in plasma Hcy levels, a nonparametric analysis of covariance (ANCOVA) was conducted using age and sex as control variables. The threshold for statistical significance was set at *P* < 0.05.

### MRI acquisition and preprocessing

Diffusion-weighted imaging data were acquired using single-shot spin-echo echo-planar sequences on a 3-T MRI unit (Tim TRIO; Siemens, Erlangen, Germany) equipped with a 40-mT/m gradient and a 32-channel receive-only phased-array head coil. Imaging parameters were as follows: echo time = 106 ms, repetition time = 5640 ms, field of view = 192 × 192 mm, 96 × 96 matrix, 70 contiguous axial slices with a thickness of 2.0 mm, 64 non-collinear diffusion weighting gradients, and a *b*-value of 1500 s/mm^2^. The *b* = 0 images were scanned three times along with two diffusion-weighted images, resulting in 69 volumes.

Source diffusion MRI data were first denoised using ‘dwidenoise’ in MRtrix3 (http://mrtrix.readthedocs.io/en/latest/index.html), by which data redundancy was exploited in the principal component analysis domain using prior knowledge of the universal Marchenko Pastur distribution in the eigenspectrum of random covariance matrices^22,23^. Subsequently, corrections were applied for eddy currents and head motion by registering all data to the first *b* = 0 image using affine transformation. Susceptibility-induced distortion was corrected through B0 unwarping using FSL 5.0.10 (http://www.fmrib.ox.ac.uk/fsl) and dti_preprocess (https:// github.com/RIKEN-BCIL/dti_preprocess). FA, axial diffusivity (AD), radial diffusivity (RD), and mean diffusivity (MD) maps were obtained by fitting a diffusion tensor model to the data using FSL DTIFIT. Among DTI measures, FA is considered the most sensitive indicator of abnormal WM integrity in SZ^24^, while AD and RD abnormalities can be indicative of axonal and myelin aberrations, respectively^25^. To analyze the FA data statistically, we applied Tract-Based Spatial Statistics (TBSS) version 1.2, a part of the FSL that allows sensitive and objective analysis of multi-subject diffusion MRI data^26^. Briefly, the TBSS process included the following steps: all FA data were spatially normalized into a common space using a nonlinear registration tool, FNIRT, a component of FSL^27,28^; the normalized FA images were averaged to create a mean FA image, which was then thinned to create an original mean FA “skeleton,” capturing the centers of WM tracts common to all subjects. This original mean FA skeleton was thresholded at an FA value of 0.2 to create the mean FA skeleton mask. The voxel values of each subject’s normalized FA map were projected onto the mean skeleton by identifying the local maxima perpendicular to the skeleton. The AD, RD, and MD maps of each subject were projected onto the skeleton using the same projection vector. The resulting skeletonized FA data were used for voxel-wise group comparisons.

### Group comparisons of FA image

Voxel-wise permutation-based nonparametric inference^29^ of FA was performed using FSL Randomize version 2.9, within a mean FA skeleton mask. People with SZ and HC were compared using ANCOVA design within a general linear model framework, with age and sex as nuisance covariates and focus on HC-minus-SZ and SZ-minus-HC. The number of permutations was 10,000. Statistical significance was set at *P* < 0.05, corrected for multiple comparisons using threshold-free cluster enhancement (TFCE)^30^. All coordinates of the local maxima within significant clusters were identified using the cluster command implemented in FSL. The fiber tracts corresponding to the clusters in the results were identified by referring to the Johns Hopkins University DTI-based White Matter Atlas (http://cmrm.med.jhmi.edu). We calculated the mean FA values for all clusters with significant between-group differences because we assumed that higher plasma Hcy would be associated with lower FA and the mean FA of the clusters with between-group differences would be more sensitive to Hcy than the mean FA of the total brain. For these clusters, we calculated the mean AD, RD, and MD values.

### Correlation analyses between Hcy and FA

The correlation between plasma Hcy levels and mean FA of the significant clusters was calculated using Spearman’s rank-order coefficients. The threshold for statistical significance was set at *P* < 0.025, with Bonferroni correction for the number of comparisons (SZ and HC groups).

### Correlation of Hcy and FA with clinical features

In the SZ group, to further examine the relationships of Hcy and FA with clinical features, we conducted Spearman’s correlation analysis of the PANSS scores and CP equivalent with plasma Hcy levels and mean FA of the significant clusters. The threshold for statistical significance was set at *P* < 0.05.

### Correlation between Hcy and FA after adjusting for age, sex, and antipsychotic medication

In the SZ group, multiple regression analysis was used to examine the potential effects of age, sex, and the CP equivalent on the correlation between plasma Hcy levels and mean FA. The threshold for significance was set at *P* < 0.05.

### Analysis after removing outliers

Using the interquartile range (IQR), 3 outliers for plasma Hcy levels (2 SZ and 1 HC participant) and 6 outliers for mean FA (5 SZ and 1 HC participant) were identified. The IQR was defined as the difference of the third quartile (Q_3_) minus the first quartile (Q_1_) of distribution, and these outliers were higher than Q_3_ plus 1.5 times the IQR or lower than Q_1_ minus 1.5 times the IQR. Therefore, we conducted additional analyses after removing these outliers. We first conducted Spearman’s correlation analysis of the plasma Hcy levels and mean FA in the SZ and HC groups. The threshold for significance was set at *P* < 0.025. Next, we conducted multiple regression analysis to examine the effects of age, sex, and the CP equivalent on the correlation between plasma Hcy levels and mean FA in the SZ group. The threshold for significance was set at *P* < 0.05.

### Assessment of microstructural composition of FA abnormality

To further examine the microstructural composition of FA abnormalities, we compared the mean AD, RD, and MD of the clusters with significant FA differences between the SZ and HC groups. In the SZ group, the correlation between plasma Hcy levels and mean AD, RD, and MD of the significant clusters was calculated using Spearman’s rank-order coefficients. The threshold for significance was set at *P* < 0.05.

### Statistics

Not all parameters were normally distributed in both the SZ and HC groups, therefore non-parametric analyses using the Mann–Whitney *U* test were performed to examine between-group differences. The threshold for significance was set at *P* < 0.05. All analyses were performed using SPSS version 29 software (SPSS Inc., Chicago, IL, USA).

## Results

### Between-group differences in plasma Hcy levels and FA images

There was no significant difference in the plasma Hcy levels between the SZ and HC groups (*P* = 0.644, Table 1). After adjusting for the effects of age and sex, there was no significant difference in the plasma Hcy levels between the SZ and HC groups (*F* (1, 134) = 0.275, *P* = 0.601).

**Table 1:** Demographic and clinical data in healthy and patient groups.

	Schizophrenia	Control	*P* Value
*N*	53	83	
Age (years)	40.38 ± 10.87	38.80 ± 10.39	0.476^a^
Sex (male/female)	27/26	44/39	0.814^b^
Homocysteine (nmol/mL)	10.48 ± 6.98	9.51 ± 3.28	0.644^a^
Mean FA of the whole clusters with significant group difference in FA	0.47 ± 0.03	0.50 ± 0.02	<0.001^a,^*
Mean AD of the whole clusters with significant group difference in FA	0.99 ± 0.04 (10^−3^)	1.00 ± 0.04 (10^−3^)	0.736^a^
Mean RD of the whole clusters with significant group difference in FA	0.48 ± 0.04 (10^−3^)	0.47 ± 0.03 (10^−3^)	0.024^a,^*
Mean MD of the whole clusters with significant group difference in FA	0.65 ± 0.04 (10^−3^)	0.64 ± 0.03 (10^−3^)	0.122^a^
CP equivalent (mg/day)	499.76 ± 362.77	–	–
PANSS total	57.79 ± 18.10	–	–
PANSS positive	13.08 ± 4.84	–	–
PANSS negative	15.87 ± 6.32	–	–
PANSS general	28.85 ± 9.06	–	–

*FA* fractional anisotropy, *AD* axial diffusivity, *RD* radial diffusivity, *MD* mean diffusivity, *CP* chlorpromazine, *PANSS* positive and negative syndrome scale, *SD* standard deviation. All data are shown as the mean ± SD.

^a^Mann–Whitney *U* test.

^b^χ^2^.

*P < 0.05.

**Fig. 1:**
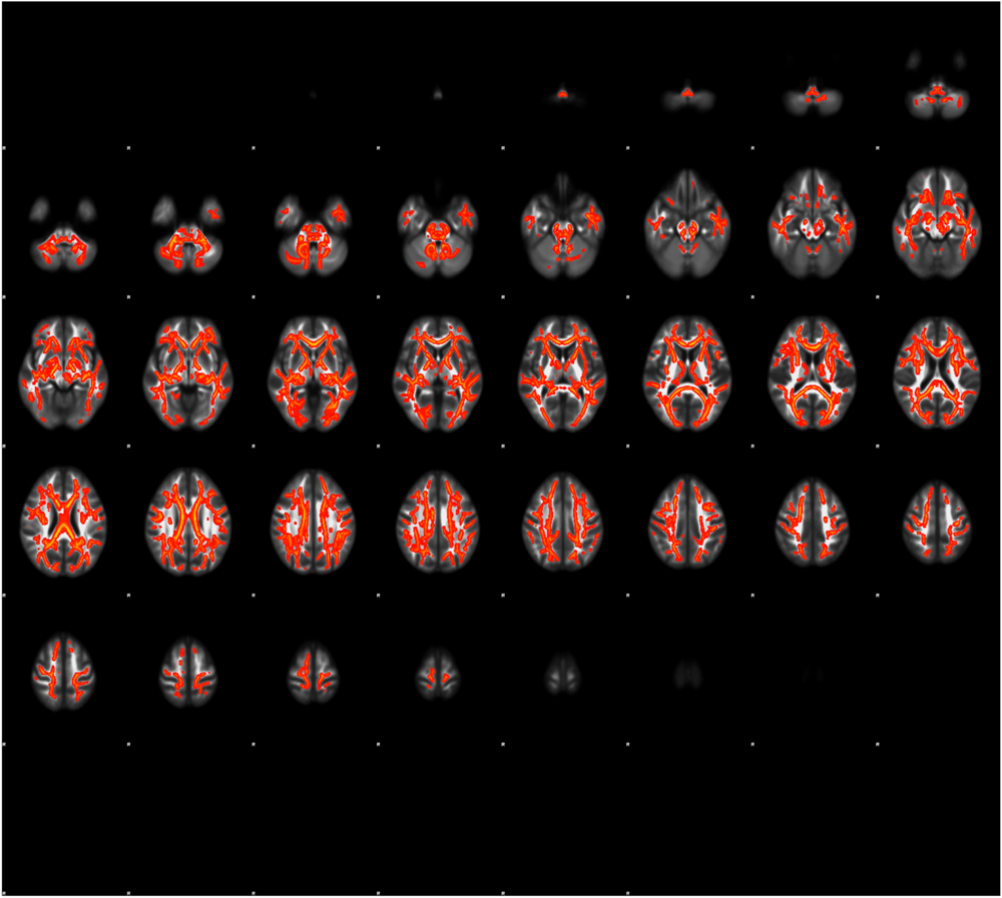
Areas with significant fractional anisotropy (FA) reduction in individuals with schizophrenia (SZ). Individuals with SZ exhibited FA reductions in areas such as the bilateral frontal, temporal, parietal, and occipital lobes, a large portion of the corpus callosum, and the corona radiata. To aid visualization, results were thickened using the tbss_fill script implemented in FSL (red-yellow). Statistical significance was set at *P* < 0.05, corrected by threshold-free cluster enhancement.

People with SZ exhibited widespread FA reduction on skeletonized FA images compared to those in the HC group. These reductions extended into the bilateral deep WM areas in the frontal, temporal, parietal, and occipital lobes, a large part of the corpus callosum, and the corona radiata (Fig. 1). There were no areas with increased FA levels in the SZ group. All significant clusters are listed in Table 2.

**Table 2:** Clusters with a significant between-group difference fractional anisotropy.

Cluster Index	Voxels	MAX X (mm)	MAX Y (mm)	MAX Z (mm)	Anatomical name of local maxima
1	64377	−19	−24	37	Left superior corona radiata

A significant cluster is shown. Voxels: the number of voxels in the cluster, MAX X/Y/Z: the location of the maximum intensity voxel in MNI (Montreal Neurological Institute) standard space coordinates (mm).

### Correlation between Hcy and FA

**Fig. 2:**
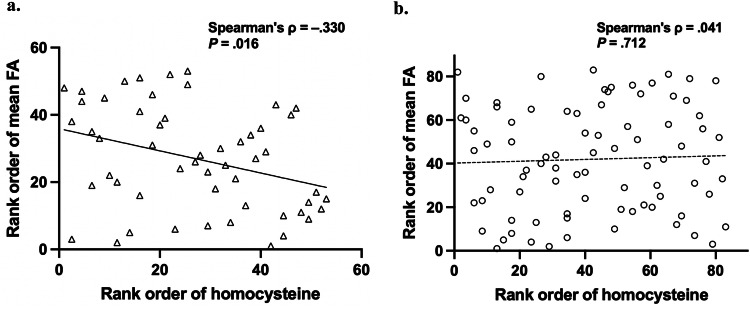
Correlation between homocysteine (Hcy) and white matter dysconnectivity. **a** The schizophrenia (SZ) group showed a statistically significant negative correlation between plasma Hcy levels and mean fractional anisotropy (FA) of the clusters with significant FA differences between the SZ and HC groups. **b** No correlation was found in healthy controls (HC). To aid visualization, Spearman’s rank orders are shown on both the X and Y axes. The regression line is shown on each graph. The filled markers and solid regression lines indicate significant results, and open markers and broken lines indicate non-significant results.

In the SZ group, there was a significant negative correlation between the plasma Hcy levels and mean FA of the clusters with a significant group difference (mean FA) (Spearman’s ρ = −0.330, *P* = 0.016 < 0.025, Fig. 2a). In contrast, no significant correlation was noted between the plasma Hcy levels and mean FA in the HC group (Spearman’s ρ = 0.041, *P* = 0.712 > 0.025, Fig. 2b).

### Correlation of Hcy and FA with clinical features

In the SZ group, there was a significant positive correlation between the plasma Hcy levels and PANSS-total score (Spearman’s ρ = .292, *P* = 0.034) and PANSS-general psychopathology subscale score (Spearman’s ρ = 0.307, *P* = 0.025) (Table 3). In contrast, no significant correlation was noted between mean FA and all PANSS scores (Table 3). Additionally, there was a significant correlation between the CP equivalent and both plasma Hcy levels (Spearman’s ρ = 0.448, *P* = 0.001) and mean FA (Spearman’s ρ = −0.350, *P* = 0.014) (Table 3).

**Table 3:** Correlations of homocysteine and mean FA with clinical features in schizophrenia.

	Homocysteine		Mean FA	
	Spearman’s ρ	*P* value	Spearman’s ρ	*P* value
PANSS total	0.292	0.034*	−0.043	0.760
PANSS positive	0.251	0.070	0.042	0.764
PANSS negative	0.210	0.131	−0.128	0.361
PANSS general	0.307	0.025*	−0.047	0.740
CP equivalent	0.448	0.001**	−0.350	0.014*

*FA* fractional anisotropy, *PANSS* positive and negative syndrome scale, *CP* chlorpromazine.

*P < 0.05, **P < 0.01.

### Correlation between Hcy and FA after adjusting for age, sex, and antipsychotic medication

In the SZ group, multiple regression analysis showed that there was no significant correlation between the plasma Hcy levels and mean FA after adjusting for age, sex, and the CP equivalent (standardized β = −0.219, *P* = 0.158, Table 4).

**Table 4:** Correlation between homocysteine and mean FA in schizophrenia.

	Unadjusted model			Adjusted model^a^		
	Standardized β	95% CI	*P* value	Standardized β	95% CI	*P* value
Homocysteine	–0.329	−0.595, −0.064	0.016*	–0.219	−0.513, 0.086	0.158
Age				−0.305	−0.586, −0.040	0.026*
Sex^b^				0.088	−0.194, 0.371	0.531
CP equivalent				–0.168	−0.467, 0.130	0.260

*FA* fractional anisotropy, *CI* confidence interval, *CP* chlorpromazine.

^a^Adjusted for age, sex, and CP equivalent.

^b^The data of sex is converted to dummy variables; male = 1, female = 2.

*P < 0.05.

### Analysis after removing outliers

In the SZ group, correlation analysis after removing these outliers also showed a significant negative correlation between the plasma Hcy levels and mean FA (Spearman’s ρ = −0.414, *P* = 0.004 < 0.025). This correlation remained significant after adjusting for age, sex, and the CP equivalent (standardized β = −0.335, *P* = 0.037, Supplementary Table 2). In contrast, no significant correlation was noted between the plasma Hcy levels and mean FA in the HC group (Spearman’s ρ = 0.047, *P* = 0.679 > 0.025).

### Assessment of microstructural composition of FA abnormality

In the SZ group, the mean RD of clusters with a notable FA difference was significantly higher in comparison to the HC group (*P* = 0.024, Table 1). No significant between-group differences in the mean AD or MD were noted (Table 1). Additionally, the plasma Hcy levels did not significantly correlate with the mean AD, RD, or MD in the SZ and HC groups (Supplementary Fig. 1).

## Discussion

To the best of our knowledge, this is the first study to demonstrate that high plasma Hcy in plasma is associated with WM microstructural disruption in people with SZ. This finding suggests that the interaction between Hcy and WM dysconnectivity could be involved in the pathophysiology of SZ. In contrast, the HC group exhibited no significant correlation between plasma Hcy levels and WM microstructural disruption. This between-group difference may be due to potential factors mediating between Hcy and WM disruption specific to people with SZ. Previous in vitro and in vivo studies showed that exposure to Hcy disrupted the WM through some pathological factors, including dysregulation of the NMDA receptor and activation of oxidative stress and neuroinflammation^14,15^. Notably, these factors also have an important role in the pathophysiology of SZ^9,31^. Thus, in people with SZ, high Hcy levels may be responsible for the WM microstructural disruption through these pathological factors. To understand the causal relationship between increased Hcy and WM dysconnectivity in SZ, further longitudinal studies investigating whether high plasma Hcy levels subsequently cause WM microstructural disruption in people with SZ are required.

We found that CP equivalent was significantly associated with plasma Hcy levels and FA reduction in people with SZ, suggesting that antipsychotic medication may be associated with increased Hcy and WM dysconnectivity in SZ. Preceding studies reported inconsistent results on the effects of antipsychotics on plasma Hcy levels in people with SZ: increased^32^, decreased^13,33^, and unchanged^34–36^. Similarly, it remains unclear whether antipsychotics reduce FA values in SZ^37–41^. However, high plasma Hcy levels and FA reductions have been reported in drug-naïve people in their first episode psychosis^13,39–45^ or those with a high risk of psychosis^46–48^, supporting that Hcy and WM dysconnectivity could be involved in the pathophysiology of SZ, independent of antipsychotic medication. In this study, after adjusting for the effects of CP equivalent, age, and sex, no significant correlation between plasma Hcy levels and mean FA was exhibited in people with SZ (standardized β = −0.219, *P* = 0.158, Table 4). Additionally, the CP equivalent was also not significantly correlated with mean FA after adjusting for the covariates (standardized β = −0.168, *P* = 0.260, Table 4). These findings suggest that neither Hcy nor antipsychotics may be directly involved in WM dysconnectivity in SZ. However, further analysis after removing outliers for plasma Hcy levels and mean FA showed different results—a significant negative correlation between plasma Hcy levels and mean FA in people with SZ (standardized β = −0.335, *P* = 0.037, Supplementary Table 2). Thus, to robustly exclude the effects of antipsychotic medication, further studies investigating the association between plasma Hcy levels and mean FA in drug-naïve people with SZ are required.

There was no significant difference in the plasma Hcy levels between the SZ and HC groups in this study. However, this finding was not consistent with a recent meta-analysis that showed increased Hcy levels in people with SZ^9^. One explanation could be for this finding in terms of the clinical severity of SZ. Prior studies showed positive correlations between plasma Hcy levels and PANSS scores^11–13^, and we also confirmed a significant positive correlation of plasma Hcy levels with PANSS-total score and PANSS-general psychopathology subscale score. These findings suggest that a population with SZ of lower clinical severity may exhibit lower plasma Hcy levels. In this study, the participants could be considered to have mild severity of SZ because the mean PANSS-total score was 57.79 ± 18.10 (Table 1), and they allowed an MRI to be performed. Thus, it is assumed that participants with mild SZ exhibited higher plasma Hcy levels than those of the HC group. It remains unclear whether an association between Hcy and WM dysconnectivity exists, regardless of the severity of SZ.

The SZ group showed widespread FA reductions on the skeletonized FA image compared with the HC group, and this was consistent with previous findings of FA reductions in SZ^1–3^. We also noted increased RD in clusters with a significant between-group difference in FA, which was indicative of myelin abnormalities^25^, and this was consistent with recent molecular studies of myelin abnormalities in people with SZ^49^. Notably, previous in vitro and in vivo studies demonstrated that exposure to Hcy leads to myelin abnormalities, and the disruption of NRG1/ErbB signaling may be one potential mechanism of the pathophysiology of SZ mediating between Hcy and myelin abnormalities in SZ. In vitro studies have shown that Hcy directly induces oligodendrocyte apoptosis^50^ or alters NRG1/ErbB signaling^51^. Additionally, an animal model study showed that loss of NRG1/ErbB signaling altered the number and morphology of oligodendrocytes and reduced myelin thickness, leading to reduced movement and social dysfunction, consistent with the clinical symptoms of SZ^52^. Thus, Hcy may trigger myelin abnormalities in SZ through altered NRG1/ErbB signaling and subsequent apoptosis in oligodendrocytes. However, in this study, the SZ group showed no significant correlation between plasma Hcy levels and mean RD (Spearman’s ρ = 0.194, *P* = 0.164, Supplementary Fig. 1c). Recently, some advanced techniques, such as myelin mapping^53,54^ and neurite orientation dispersion and density imaging (NODDI)^55–57^, have been performed to estimate whether FA abnormality is derived from myelin or axons because DTI cannot robustly confirm this. Thus, to further examine the relationship between Hcy and myelin abnormalities in SZ, myelin mapping should be performed in the future.

Our findings have important clinical implications for SZ treatment. Betaine serves as the primary therapy for homocystinuria^58^ by converting Hcy to methionine^59^ and confers neuroprotective effects against demyelination by increasing the S-adenosylmethionine/S-adenosylhomocysteine (SAM/SAH) ratio in a cuprizone mouse model^60^. Furthermore, betaine ameliorates schizophrenic behaviors in mice^61,62^, and an open-label clinical trial showed that betaine supplementation improved the PANSS-positive subscale score in people with SZ^63^. These findings suggest that betaine may have potential therapeutic effects in targeting an interaction between Hcy and WM disruption in SZ, and our findings may also support the important role of this interaction in the pathophysiology of SZ.

We previously reported an association between FA reduction and carbonyl stress^19^, an abnormal metabolic condition resulting from elevated levels of reactive carbonyl compounds (RCOs)^64^. We further explored the association between plasma Hcy levels and carbonyl stress markers, including plasma pentosidine and serum pyridoxal levels^19^, and found no significant correlation between Hcy and carbonyl stress markers in either the SZ or HC groups. Thus, this finding suggests that Hcy may be associated with WM dysconnectivity independent of carbonyl stress.

This study had some limitations. First, we could not investigate the effects of several potential factors on Hcy metabolism, such as serum folic acid and vitamin B12 levels and *MTHFR* C677T polymorphism^65^. Subsequent research should control for these variables to further examine how Hcy is associated with WM microstructural disruption in SZ. Second, previous studies have shown that FA reduction is associated with cognitive impairment in people with SZ^4–6^, and we could not show an association of the mean FA with the clinical severity of SZ, assessed using the PANSS (Table 3). Thus, further studies using cognitive function scales are required to examine the detailed clinical features of people with high Hcy levels and FA reductions. Finally, this cross-sectional study mandates further longitudinal research to confirm a causal relationship between increased Hcy and WM dysconnectivity in the pathophysiology of SZ.

## Conclusion

Herein, we noted a negative correlation between plasma Hcy levels and FA values using DTI in people with SZ, suggesting that increased Hcy could be associated with WM dysconnectivity in SZ. This finding reinforces the dysconnectivity hypothesis of SZ; an interaction between Hcy and WM dysconnectivity could be a potential mechanism of the pathophysiology of SZ. Further, longitudinal studies are required to investigate whether high plasma Hcy levels subsequently cause FA reduction in people with SZ.

### Supplementary information

Supplemental Material

Supplemental Table 1

## Data Availability

The data described in the manuscript, codebook, and analytical code will be made available upon request.

## References

[1] Kelly S (2018). Widespread white matter microstructural differences in schizophrenia across 4322 individuals: results from the ENIGMA Schizophrenia DTI Working Group. Mol. Psychiatry.

[2] Cetin-Karayumak S (2020). White matter abnormalities across the lifespan of schizophrenia: a harmonized multi-site diffusion MRI study. Mol. Psychiatry.

[3] Barth C (2023). In vivo white matter microstructure in adolescents with early-onset psychosis: a multi-site mega-analysis. Mol. Psychiatry.

[4] Holleran L (2020). The relationship between white matter microstructure and general cognitive ability in patients with schizophrenia and healthy participants in the ENIGMA consortium. Am. J. Psychiatry.

[5] Seitz-Holland J (2022). Cognitive deficits, clinical variables, and white matter microstructure in schizophrenia: a multisite harmonization study. Mol. Psychiatry.

[6] Kochunov P (2017). Association of white matter with core cognitive deficits in patients with schizophrenia. JAMA Psychiatry.

[7] Muntjewerff JW, Kahn RS, Blom HJ, den Heijer M (2006). Homocysteine, methylenetetrahydrofolate reductase and risk of schizophrenia: a meta-analysis. Mol. Psychiatry.

[8] Nishi A (2014). Meta-analyses of blood homocysteine levels for gender and genetic association studies of the MTHFR C677T polymorphism in schizophrenia. Schizophr. Bull..

[9] Fraguas D (2019). Oxidative stress and inflammation in first-episode psychosis: a systematic review and meta-analysis. Schizophr. Bull..

[10] Numata S (2015). Evaluation of an association between plasma total homocysteine and schizophrenia by a Mendelian randomization analysis. BMC Med. Genet..

[11] Yang X (2022). Increased serum homocysteine in first episode and drug-naïve individuals with schizophrenia: sex differences and correlations with clinical symptoms. BMC Psychiatry.

[12] Misiak B, Frydecka D, Slezak R, Piotrowski P, Kiejna A (2014). Elevated homocysteine level in first-episode schizophrenia patients—the relevance of family history of schizophrenia and lifetime diagnosis of cannabis abuse. Metab. Brain Dis..

[13] Fan N (2017). Effect of risperidone on serum homocysteine levels in first-episode, drug-naïve patients with schizophrenia. Neurosci. Lett..

[14] Moustafa AA, Hewedi DH, Eissa AM, Frydecka D, Misiak B (2014). Homocysteine levels in schizophrenia and affective disorders-focus on cognition. Front. Behav. Neurosci..

[15] Smith AD, Refsum H (2016). Homocysteine, B vitamins, and cognitive impairment. Annu. Rev. Nutr..

[16] Zhou H (2022). Elevated homocysteine levels, white matter abnormalities and cognitive impairment in patients with late-life depression. Front. Aging Neurosci..

[17] Lee CC (2017). Effects of homocysteine on white matter diffusion parameters in Alzheimer’s disease. BMC Neurol..

[18] Sampedro F (2022). Increased homocysteine levels correlate with cortical structural damage in Parkinson’s disease. J. Neurol. Sci..

[19] Son S (2020). Enhanced carbonyl stress and disrupted white matter integrity in schizophrenia. Schizophr. Res..

[20] Inada T, Inagaki A (2015). Psychotropic dose equivalence in Japan. Psychiatry Clin. Neurosci..

[21] Lehman AF (2004). Practice guideline for the treatment of patients with schizophrenia, second edition. Am. J. Psychiatry.

[22] Veraart J, Fieremans E, Novikov DS, Diffusion MRI (2016). noise mapping using random matrix theory. Magn. Reson. Med..

[23] Veraart J (2016). Denoising of diffusion MRI using random matrix theory. Neuroimage.

[24] Ellison-Wright I, Bullmore E (2009). Meta-analysis of diffusion tensor imaging studies in schizophrenia. Schizophr. Res..

[25] Seal ML (2008). Abnormal white matter microstructure in schizophrenia: a voxelwise analysis of axial and radial diffusivity. Schizophr. Res..

[26] Smith SM (2006). Tract-based spatial statistics: voxelwise analysis of multi-subject diffusion data. Neuroimage.

[27] Andersson, J. L. R., Jenkinson, M. & Smith, S. Non-linear optimisation. FMRIB Technical Report TR07JA1. *FMRIB Centre* (2007).

[28] Andersson JLR, Jenkinson M, Smith S (2007). Nonlinear registration, aka spatial normalisation. FMR1B Technical Report TR07JA2. FMRIB Analysis Group of the University of Oxford.

[29] Genovese CR, Lazar NA, Nichols T (2002). Thresholding of statistical maps in functional neuroimaging using the false discovery rate. Neuroimage.

[30] Smith SM, Nichols TE (2009). Threshold-free cluster enhancement: addressing problems of smoothing, threshold dependence and localisation in cluster inference. Neuroimage.

[31] McCutcheon RA, Krystal JH, Howes OD (2020). Dopamine and glutamate in schizophrenia: biology, symptoms and treatment. World Psychiatry.

[32] Misiak B, Frydecka D, Łaczmański Ł, Ślęzak R, Kiejna A (2014). Effects of second-generation antipsychotics on selected markers of one-carbon metabolism and metabolic syndrome components in first-episode schizophrenia patients. Eur. J. Clin. Pharmacol..

[33] Petronijević ND (2008). Plasma homocysteine levels in young male patients in the exacerbation and remission phase of schizophrenia. Prog. Neuropsychopharmacol. Biol. Psychiatry.

[34] Hidalgo-Figueroa M (2023). Association of prolactin, oxytocin, and homocysteine with the clinical and cognitive features of a first episode of psychosis over a 1-year follow-up. Int. J. Neuropsychopharmacol..

[35] Baeza I (2017). The effects of antipsychotics on weight gain, weight-related hormones and homocysteine in children and adolescents: a 1-year follow-up study. Eur. Child Adolesc. Psychiatry.

[36] Wysokiński A, Kłoszewska I (2013). Homocysteine levels in patients with schizophrenia on clozapine monotherapy. Neurochem. Res..

[37] Bergé D (2020). Elevated extracellular free-water in a multicentric first-episode psychosis sample, decrease during the first 2 years of illness. Schizophr. Bull..

[38] Barth C (2020). Exploring white matter microstructure and the impact of antipsychotics in adolescent-onset psychosis. PLoS One.

[39] Serpa MH (2017). State-dependent microstructural white matter changes in drug-naïve patients with first-episode psychosis. Psychol. Med..

[40] Zeng B (2016). Abnormal white matter microstructure in drug-naive first episode schizophrenia patients before and after eight weeks of antipsychotic treatment. Schizophr. Res..

[41] Wang Q (2013). White-matter microstructure in previously drug-naive patients with schizophrenia after 6 weeks of treatment. Psychol. Med..

[42] Kraguljac NV (2021). White matter integrity, duration of untreated psychosis, and antipsychotic treatment response in medication-naïve first-episode psychosis patients. Mol. Psychiatry.

[43] Zeng J (2021). Pretreatment abnormalities in white matter integrity predict one-year clinical outcome in first episode schizophrenia. Schizophr. Res..

[44] Zhang Q (2021). Unveiling the metabolic profile of first-episode drug-naïve Schizophrenia patients: baseline characteristics of a longitudinal study among Han Chinese. Front. Psychiatry.

[45] Onozato M (2020). Alterations in methionine to homocysteine ratio in individuals with first-episode psychosis and those with at-risk mental state. Clin. Biochem..

[46] Tang Y (2019). Altered cellular white matter but not extracellular free water on diffusion MRI in individuals at clinical high risk for psychosis. Am. J. Psychiatry.

[47] Katagiri N (2015). A longitudinal study investigating sub-threshold symptoms and white matter changes in individuals with an ‘at risk mental state’ (ARMS). Schizophr. Res..

[48] Karlsgodt KH, Niendam TA, Bearden CE, Cannon TD (2009). White matter integrity and prediction of social and role functioning in subjects at ultra-high risk for psychosis. Biol. Psychiatry.

[49] Cassoli JS (2015). Disturbed macro-connectivity in schizophrenia linked to oligodendrocyte dysfunction: from structural findings to molecules. npj Schizophr..

[50] Pak KJ, Chan SL, Mattson MP (2003). Homocysteine and folate deficiency sensitize oligodendrocytes to the cell death-promoting effects of a presenilin-1 mutation and amyloid beta-peptide. Neuromolecular Med..

[51] Qin XF, Shan YG, Dou M, Li FX, Guo YX (2023). Notch1 signaling activation alleviates coronary microvascular dysfunction through histone modification of Nrg‐1 via the interaction between NICD and GCN5. Apoptosis.

[52] Roy K (2007). Loss of erbB signaling in oligodendrocytes alters myelin and dopaminergic function, a potential mechanism for neuropsychiatric disorders. Proc. Natl. Acad. Sci. USA.

[53] Glasser MF (2022). Empirical transmit field bias correction of T1w/T2w myelin maps. Neuroimage.

[54] Wei W (2022). Structural covariance of depth-dependent intracortical myelination in the human brain and its application to drug-naïve schizophrenia: a T1w/T2w MRI study. Cereb. Cortex.

[55] Zhang H, Schneider T, Wheeler-Kingshott CA, Alexander DC (2012). NODDI: practical in vivo neurite orientation dispersion and density imaging of the human brain. Neuroimage.

[56] Rae CL (2017). Deficits in neurite density underlie white matter structure abnormalities in first-episode psychosis. Biol. Psychiatry.

[57] Kraguljac NV (2019). A longitudinal neurite and free water imaging study in patients with a schizophrenia spectrum disorder. Neuropsychopharmacology.

[58] Gerrard A, Dawson CJ (2022). Homocystinuria diagnosis and management: it is not all classical. J. Clin. Pathol..

[59] Ueland PM (2011). Choline and betaine in health and disease. J. Inherit. Metab. Dis..

[60] Singhal NK (2020). Betaine restores epigenetic control and supports neuronal mitochondria in the cuprizone mouse model of multiple sclerosis. Epigenetics.

[61] Ohnishi T (2019). Investigation of betaine as a novel psychotherapeutic for schizophrenia. EBioMedicine.

[62] Yoshihara S (2021). Betaine ameliorates schizophrenic traits by functionally compensating for KIF3-based CRMP2 transport. Cell Rep..

[63] Kirihara K (2022). Betaine supplementation improves positive symptoms in schizophrenia. Schizophr. Res..

[64] Miyata T, van Ypersele de Strihou C, Kurokawa K, Baynes JW (1999). Alterations in nonenzymatic biochemistry in uremia: origin and significance of “carbonyl stress” in long-term uremic complications. Kidney Int..

[65] Sun C, Ding D, Wen Z, Zhang C, Kong J (2023). Association between micronutrients and hyperhomocysteinemia: a case-control study in Northeast China. Nutrients.

