# Supplementary material for: Association of homocysteine with white matter dysconnectivity in schizophrenia
Source: Schizophrenia (Heidelb). 2024 Mar 20;10(1):39. doi: 10.1038/s41537-024-00458-0 (PMC10954654; doi:10.1038/s41537-024-00458-0)
Supplement: Supplementary file 1 — Supplemental Material [file 41537_2024_458_MOESM1_ESM.pdf]

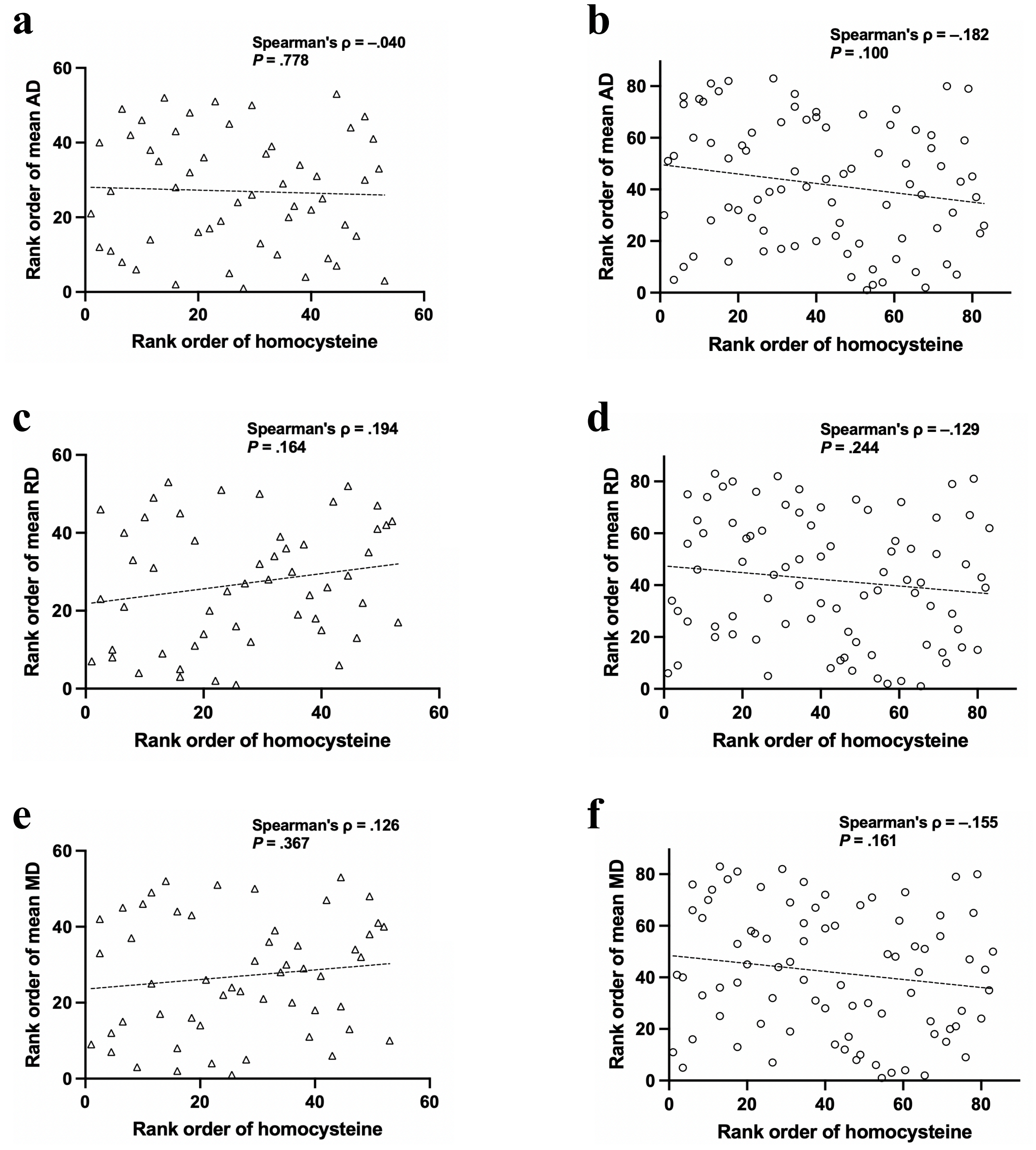

Supplemental Figure 1. Correlations of homocysteine with the microstructural compositions of fractional anisotropy (FA) abnormalities. a) b) The schizophrenia (SZ) and the healthy controls (HC) showed no significant correlation between plasma homocysteine levels and mean axial diffusivity (AD) of the clusters with significant FA

differences between the SZ and HC groups. c) d) No correlation was found between plasma Hcy levels and mean radial diffusivity (RD) of the clusters. e) f) No correlation was found between plasma Hcy levels and mean mean diffusivity (MD) of the clusters. To aid visualization, Spearman’s rank orders are shown on both the X and Y axes. The regression line is shown on each graph, and open markers and broken lines indicate non-significant results.

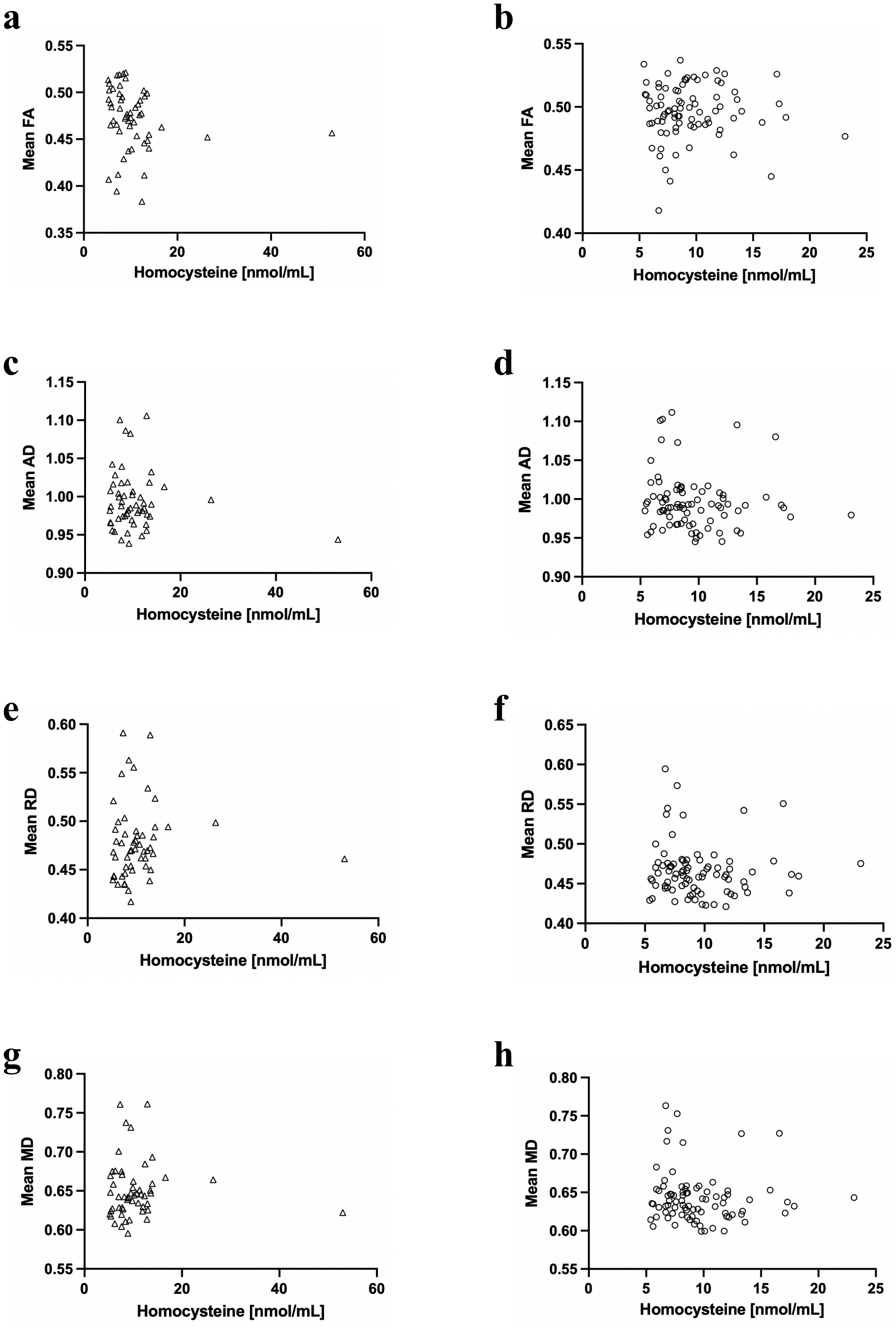
a
Mean FA
0.55
0.50
0.45
0.40
0.35
0
20
40
60
Homocysteine [nmol/mL]
b
Mean FA
0.55
0.50
0.45
0.40
0
5
10
15
20
25
Homocysteine [nmol/mL]
c
Mean AD
1.15
1.10
1.05
1.00
0.95
0.90
0
20
40
60
Homocysteine [nmol/mL]
d
Mean AD
1.15
1.10
1.05
1.00
0.95
0.90
0
5
10
15
20
25
Homocysteine [nmol/mL]
e
Mean RD
0.60
0.55
0.50
0.45
0.40
0
20
40
60
Homocysteine [nmol/mL]
f
Mean RD
0.65
0.60
0.55
0.50
0.45
0.40
0
5
10
15
20
25
Homocysteine [nmol/mL]
g
Mean MD
0.80
0.75
0.70
0.65
0.60
0.55
0
20
40
60
Homocysteine [nmol/mL]
h
Mean MD
0.80
0.75
0.70
0.65
0.60
0.55
0
5
10
15
20
25
Homocysteine [nmol/mL]

Supplemental Figure 2. Scatterplots of homocysteine (Hcy) and white matter dysconnectivity. Original values are shown on both the X and Y axes. a) b) Plasma Hcy levels and mean fractional anisotropy (FA) of the clusters in the SZ and HC groups. c) d) Plasma Hcy levels and mean axial diffusivity (AD) of the clusters in the SZ and HC groups. e) f) Plasma Hcy levels and mean radial diffusivity (RD) of the clusters in the SZ and HC groups. g) h) Plasma Hcy levels and mean mean diffusivity (MD) of the clusters in the SZ and HC groups.

Supplemental Table 2

Correlation between homocysteine and mean FA after removing outliers in schizophrenia.

	Unadjusted model			Adjusted model [a]		
	Standardized β	95%CI	*P* value	Standardized β	95%CI	*P* value
Homocysteine	–.413	−.690, −.136	.004**	−.335	−.614, −.020	.037*
Age				−.215	−.496, .065	.127
Sex [b]				.076	−.207, .355	.596
CP equivalent				−.217	−.514, .091	.164

FA fractional anisotropy, *CI* confidence interval, *CP* chlorpromazine.

[a] Adjusted for age, sex, and CP equivalent.

[b] The data of sex is converted to dummy variables; male = 1, female = 2.

* $P < .05$, ** $P < .01$.
